# Towards the Automatic Localization of the Irritative Zone Through Magnetic Source Imaging

**DOI:** 10.1007/s10548-020-00789-y

**Published:** 2020-08-07

**Authors:** Gianvittorio Luria, Dunja Duran, Elisa Visani, Davide Rossi Sebastiano, Alberto Sorrentino, Laura Tassi, Alice Granvillano, Silvana Franceschetti, Ferruccio Panzica

**Affiliations:** 1 Department of Neurophysiology and Diagnostic Epileptology, IRCCS Foundation Carlo Besta Neurological Institute, Milan, Italy; 2 Department of Mathematics, University of Genoa, Genoa, Italy; 3 CNR - SPIN, Genoa, Italy; 4 Epilepsy Surgery Center, Ospedale Niguarda, Milan, Italy

**Keywords:** Dipole modeling, Bayesian methods, Magnetic source imaging, Epilepsy, Magnetoencephalography

## Abstract

The present work aims at validating a Bayesian multi-dipole modeling algorithm (SESAME) in the clinical scenario consisting of localizing the generators of single interictal epileptiform discharges from resting state magnetoencephalographic recordings. We use the results of Equivalent Current Dipole fitting, performed by an expert user, as a benchmark, and compare the results of SESAME with those of two widely used source localization methods, RAP-MUSIC and wMNE. In addition, we investigate the relation between post-surgical outcome and concordance of the surgical plan with the cerebral lobes singled out by the methods. Unlike dipole fitting, the tested algorithms do not rely on any subjective channel selection and thus contribute towards making source localization more unbiased and automatic. We show that the two dipolar methods, SESAME and RAP-MUSIC, generally agree with dipole fitting in terms of identified cerebral lobes and that the results of the former are closer to the fitted equivalent current dipoles than those of the latter. In addition, for all the tested methods and particularly for SESAME, concordance with surgical plan is a good predictor of seizure freedom while discordance is not a good predictor of poor post-surgical outcome. The results suggest that the dipolar methods, especially SESAME, represent a reliable and more objective alternative to manual dipole fitting for clinical applications in the field of epilepsy surgery.

## Introduction

Epilepsy is a neurological disorder affecting 50 million people worldwide (World Health Organization et al. 2019). Of those, about 30% fail to respond to anti-epileptic drugs (Eadie 2012; Tavakol et al. 2019) and, when diagnosed with focal seizure onset, might resort to resective or disconnective surgery, provided that the supposed Epileptogenic Zone (EZ) is identified (Jehi 2018). In most cases, the localization of the EZ is achieved by means of routine electro-clinical investigations and imaging methods, such as semiology, ElectroEncephaloGraphy (EEG) and Magnetic Resonance Imaging (MRI), leading to good seizure outcome after surgery. For about 30% of surgical candidates, however, the electro-clinical data yield discrepant outcomes and/or the MRI is contradictory or unrevealing. In such cases, invasive monitoring of the supposed EZ through implantation of Stereo-ElectroEncephaloGraphic (SEEG) electrodes becomes necessary (Cossu et al. 2005; Cardinale et al. 2012), and RadioFrequency THermoCoagulation (RF-THC) can be performed during SEEG recordings. However, in this scenario, despite the use of invasive pre-surgical techniques, surgery frequently does not lead to seizure freedom, with up to 40% of patients suffering from seizure relapses, regardless of age, gender and cerebral lobe affected by epilepsy (Téllez-Zenteno et al. 2005; Kim et al. 2017). On this account, non-invasive functional neuroimaging techniques, such as MagnetoEncephaloGraphy (MEG), high-resolution EEG, positron emission tomography, single photon emission computed tomography and EEG-fMRI, have been proposed for the identification of the EZ and are expected to avoid or to guide the SEEG exploration. Among these techniques, MEG is increasingly used, mainly for its excellent temporal resolution combined with a good spatial resolution. In this regard, it has been shown that magnetic source imaging (MSI) has clinical value in predicting seizure-free surgical outcome in epilepsy surgery (Knowlton et al. 2008; Carrette and Stefan 2019). MEG recordings of epileptic patients are mostly used to determine the Irritative Zone (IZ), i.e. the cortical area where Interictal Epileptiform Discharges (IEDs) originate. It has been already reported in the literature that the IZ represents a valid surrogate for the EZ localization, since IEDs-based analysis agrees with information on the zone of seizure origin derived from permanently implanted intracranial electrodes (Hufnagel et al. 2000; Pittau et al. 2014).

The standard approach to IZ localization from MEG data comprises (i) data cleaning, (ii) IEDs identification in the MEG signal, (iii) possibly some form of data averaging to increase the signal-to-noise ratio (SNR) and (iv) source localization at selected time points. Despite the availability of a multitude of inverse source localization methods, Equivalent Current Dipole (ECD) fitting (Merlet and Gotman 1999) remains the most widely used (Mouthaan et al. 2016; Hari et al. 2018) and the only one recommended by the American Clinical Magnetoencephalography Society (Bagic et al. 2011; Carrette and Stefan 2019). This seems to be a reasonable choice, particularly because some studies showed that dipole fitting estimation was more accurate than distributed source techniques (Duez et al. 2019). On the other hand, dipole localization from MEG data is itself a time-consuming and complex procedure involving subjective choices, and therefore reliable only when performed by experienced users.

In this work we provide a contribution towards the automation of dipole source modeling in the context of IZ localization, by validating an analysis pipeline, based on the Bayesian multi-dipole estimation method SESAME (Sorrentino et al. 2014; Sommariva and Sorrentino 2014), which automatically reproduces results comparable with those obtained by expert users with manual dipole fitting. SESAME is an iterative Monte Carlo algorithm that approximates the posterior distribution for an a-priori unknown number of dipoles; it provides posterior probability for different number of sources, a posterior probability cortical map and estimates of locations and time courses of each dipole. Here we used SESAME to estimate single dipoles at specific time points corresponding to the peaks of individual IEDs.

To reflect the variability of clinical cases, the validation of SESAME was performed on clinical data involving patients with focal drug-resistant epilepsy with two different conditions: MRI-negative patients and patients in which a cortical lesion visible on MRI was supposed to be the cause of the epilepsy. For both groups of patients, we used as a benchmark the results obtained by an ECD fitting analysis performed by an expert neurophysiologist. Moreover, we compared the results provided by SESAME with those obtained with two other well-established automatic source localization methods, namely Recursively Applied and Projected MUltiple SIgnal Classification (RAP-MUSIC) and weighted Minimum Norm Estimate (wMNE). RAP-MUSIC (Mosher and Leahy 1999) is an automatic multi-dipole reconstruction method, in which the number of dipoles must be set in advance by the user. wMNE (Lin et al. 2006) is probably the most widely used inverse method based on a distributed source model; it is a weighted version of classical MNE, where the weighting aims at removing the bias towards superficial sources, typical of classical MNE.

There is still much debate on whether to apply source modeling to single IEDs or to the averages of multiple IEDs, and how to interpret the variability of source locations estimated from different single IEDs. According to Bast et al. (2006), for instance, such variability is largely due to the low SNR of the data. On the other hand, in Bouet et al. (2012) the authors claim that using single IEDs yields a better characterization of the extent of the IZ, at the price of working with lower SNR data. Here, in agreement with Bouet et al. (2012), we chose to work with single IEDs, thus also providing a stronger validation of our analysis pipeline. Indeed, while in the ECD fitting analysis the low SNR is substantially mitigated by the channel selection performed by the expert user, this does not happen for the automatic source localization methods which were applied to the whole signal, thus making the automatic localization more challenging.

## Materials and Methods

### Patients

Twenty-two patients with drug-resistant focal epilepsy, eligible to epilepsy surgery, were consecutively enrolled for this analysis. Among them, nine patients showed cortical lesion on MRI images, while thirteen patients were MRI-negative.

All patients underwent a MEG recording after a comprehensive electro-clinical and MRI evaluation. Furthermore, in twelve out of the twenty-two patients enrolled, a pre-surgical invasive assessment by means of SEEG was performed.

The eligibility for epilepsy surgery and surgical plan was decided after comprehensive discussions involving the referring neurologist, epileptologists, neurosurgeons, and neuroradiologists, blind to MEG results. All of the resections were performed for strictly therapeutic reasons; the extent of the excision was planned preoperatively on the basis of the supposed EZ location and of the risk of post-surgical deficits. Post-surgical outcome was evaluated in all the patients at least one year after surgery according with the Engel scale (Engel Jr 1993). Clinical data are reported in Table 1.

All the procedures and protocols have been approved by the Ethical Committees of the involved institutions and performed after written informed consent from all patients.

**Table 1:** Clinical data

ID	Gender	Age	# IED	SEEG	MRI	RF-THC	Surgery	Engel Class
P1	F	25	36	✗	R F/C FCD	✗	R F	3
P2	M	47	30	✗	L P FCD	✗	L P	1
P3	M	56	61	✗	L T FCD	✗	L T	1
P4	F	31	92	✗	L T G	✗	L T	4
P5	F	25	18	✗	L T FCD	✗	L T	1
P6	M	24	41	✗	R F FCD	✗	R F	1
P7	F	16	8	✗	L T G	✗	L T	2/3
P8	M	27	14	✗	R/L T/P U	✗	R T/P	1
P9	F	19	100	✗	R P FCD	✗	✗	✗
P10	F	26	45	✗	Negative	✗	✗	✗
P11	M	21	75	L F	Negative	✓	L F	1
P12	M	20	35	R F	Negative	✗	R F	1
P13	M	24	47	L T/O	Negative	✗	L T/O	1
P14	F	21	17	L P	Negative	✓	L P	2
P15	F	24	39	R C/P	Negative	✗	R C/P	1
P16	F	33	62	R T	Negative	✓	✗	1
P17	M	33	52	R T	Negative	✗	R T	1
P18	F	21	12	R T/O	Negative	✓	R T/O	1
P19	F	27	52	R F/T/P	Negative	✓	R F/T	4
P20	M	44	72	L T	Negative	✓	L T	2
P21	M	21	64	R T/P/O	Negative	✓	R T/P/O	1
P22	F	36	82	L C/T/P	Negative	✓	L T	3

Columns represent: Gender, Age, number of selected IEDs, StereoEEG, MRI, Radio Frequency THermoCoagulation, Surgery and Engel Class

*L* left, *R* right, *F* frontal, *C* central; *P* parietal, *T* temporal, *O* occipital, *FCD* focal cortical dysplasia, *G* glioma, *GG* ganlioglioma, *U* ulegyria

### Data Acquisition

MEG recordings were acquired at a sampling rate of 1 kHz using a 306-channel whole-head neuromagnetometer (Triux, Elekta Oy, Helsinki, Finland) for about 60 minutes at rest. The subject’s head position inside the MEG helmet was continuously monitored by five head position identification coils located on the scalp. The locations of these coils, together with three anatomical landmarks (nasion, right and left preauriculars), and additional scalp points were digitized before the recording by means of a 3D digitizer (FASTRAK, Polhemus, Colchester, VT). The scalp surface points were used for the co-registration with the patient’s anatomical MRI. The raw MEG data were pre-processed off-line with the temporally extended Signal Space Separation method (tSSS) implemented in the Maxfilter 2.2 (Elekta Neuromag Oy, Helsinki, Finland) to suppress external interferences and correct for head movements (Taulu and Hari 2009), and next filtered at 0.1–100 Hz.

MRI images were acquired by means of a volumetric T1-weighted sequence on a 3T MR scanner (Philips Healthcare BV, Best, NL).

### Source Modeling

Before application of source modeling methods, a pre-processing step was applied in order to clean the data. Specifically, data were first bandpass filtered with a 1 Hz highpass (with 1 Hz transition band) and a 40 Hz lowpass (with 10 Hz transition band); then physiological artifacts (such as heart beats and eye blinks) were removed by means of visual inspection of topographies and time series of individual components after Independent Component Analysis. Only gradiometer channels were selected from the MEG recordings.

MEG signals were visually inspected for IEDs by an expert neurophysiologist, using the criteria suggested by Enatsu et al. (2008). For each patient the most frequent IEDs of similar morphology were selected. Source modeling of individual topographies, each corresponding to the peak of a selected IED, was then performed by means of the following methods: single ECD fit; Bayesian multi-dipole modeling with SESAME; dipole estimation with RAP-MUSIC; distributed source estimation with wMNE.

For MRI-negative patients, a cortical source space was set up, containing on average 8195 points and with an approximate source spacing of 4.9 mm, with small differences among subjects; for patients with cortical lesion, a volume source space was instead used, with 5 mm spacing between neighbouring points. The forward solution was computed by means of a single-layer Boundary Element Method (BEM) with standard conductivity equal to 0.3 S/m. The same leadfields were used for all methods. The simplified single-layer BEM model is justified by the fact that, generally speaking, just a brain-shaped homogeneous conductor is sufficient for the computation of the magnetic field (Hamalainen and Sarvas 1989). However, since the realistic geometry of head tissue was used, existence of tissue inhomogeneities may have introduced secondary current sources (Schomer and Da Silva 2012) which may have affected differently the performance of each method.

ECDs were estimated from a subset of sensors around the one that showed the highest amplitude IED. The number of selected channels was variable, and was chosen to enhance the localization of the signal of interest. The statistical criteria for defining the localization were the following: goodness of fit greater than or equal to 80%, confidence volume less than 1000 mm$^3$ and dipole moment between 50 and 500 nAm. The ECD analysis was performed with Elekta Neuromag Xfit software.

SESAME is an iterative method that provides increasingly complex solutions, i.e. solutions with an increasing number of dipoles, as the iterations advance. In principle, one would stop the iterative procedure when the discrepancy between the measured and the predicted data reaches a given threshold, corresponding to an estimate of the noise level. In this study, however, we are explicitly looking for a single area, and therefore we stop the procedure at the last iteration where a single dipole is estimated. As explained in Sorrentino et al. (2014), this corresponds to an adaptive choice of the noise standard deviation. SESAME was applied with 100 Monte Carlo samples. The other parameter, namely the standard deviation of the Gaussian prior on the dipole moment, was set as the ratio between the maximum of the data and the maximum of the leadfield.

We used the Python implementation of SESAME available at https://github.com/pybees/sesameeg. For both RAP-MUSIC and wMNE we used the MNE-Python package (Gramfort et al. 2013, 2014). In RAP-MUSIC, the number of dipoles was set to one. wMNE was applied with free orientation, and with the standard, automatically computed depth-weighting.

### Performance Evaluation

Before proceeding with the description of the performance metrics, we recall what the output of the three used methods are. The output of SESAME is a posterior distribution for a variable number of dipoles and their parameters. From this distribution, a cortical probability map is computed, quantifying for each voxel the posterior probability of containing a dipolar source; in addition, a point estimate of the dipole location is worked out as the peak of the cortical probability map. The output of RAP-MUSIC is a single current dipole. Finally, the output of wMNE is a cortical intensity map, quantifying how strong the estimated electrical current at each voxel is; from this distribution, an estimate of dipole location is computed as the peak of the intensity map.

Evaluation of the performance of the source modeling methods has been based on the results of the ECD fitting analysis, taken here as a benchmark, and has been quantified by means of four metrics: the Dipole Localization Discrepancy (DLD), the Map Localization Discrepancy (MLD), the Spatial Dispersion (SD) and the Area Under the Curve (AUC).

**Fig. 1:**
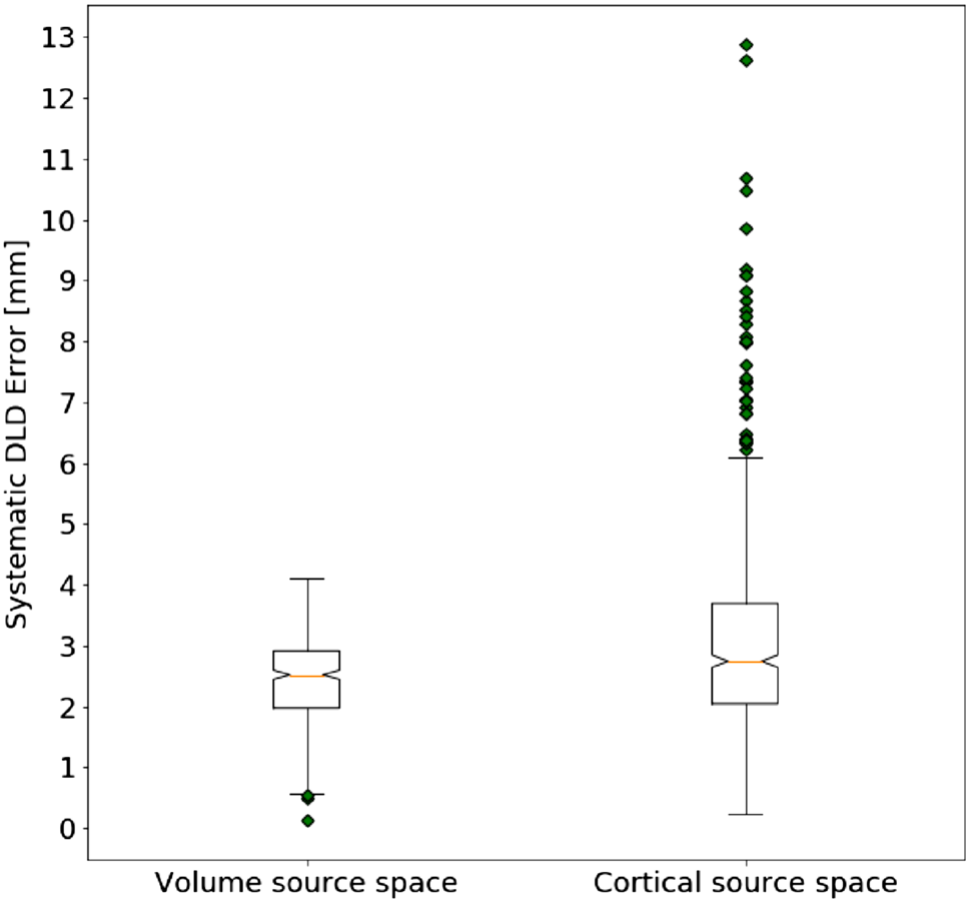
Boxplots of the distance between the ECD locations and the closest grid point, for all ECDs and all patients; for the volume source space (left), the maximum distance is less than 5 mm; for the cortical source space (right), which is not homogeneous, the maximum distance goes up to 13 mm. We can consider the distance of 2.65 mm as an average systematic error affecting the DLD

The DLD is the Euclidean distance between the ECD location and the source position estimated by the automatic methods; it evaluates only the quality of the point estimate. This metric is affected by a systematic error, to the extent that ECD locations can belong to any point in space while the three automated methods use a discretized source space, i.e. estimated dipole locations belong to a finite grid; in Fig. 1 we present the boxplots of the distances between each estimated ECD location and its nearest grid point; in doing so, we distinguish between volume source space and cortical source space because the latter presents more outliers, in the presence of ECD locations falling relatively far from the possibly imperfect discretization of the cortical surface. For the volume source space (400 ECDs) the median is 2.52 mm, while it is 2.74 mm for the cortical source space (654 ECDs); we can thus consider 2.65 mm as an average systematic error affecting the DLD. This metric can be used to evaluate the performance of each tested method.

The MLD (Molins et al. 2008) is defined as

1 $$\begin{aligned} \text {MLD} := \sqrt{\frac{\sum _{j=1}^{N_v} \big (d_j\ |S_j|\big )^2}{\sum _{j=1}^{N_v}|S_j|^2}}\ , \end{aligned}$$

where $N_v$ is the number of voxels, $d_j$ is the distance between the *j*-th voxel and the ECD location and $S_j$ is the value of the cortical map at the *j*-th voxel. The MLD evaluates the discrepancy between the cortical map and the ECD location: it weights the distance between the voxel and the ECD with the weight $S_j$ of the voxel itself, thus penalizing both distributions that are highly peaked in a wrong voxel, and distributions that are highly dispersed. The MLD is affected by the same systematic error as the DLD. This metric cannot be computed for ECD fitting nor for RAP-MUSIC, since these methods do not output any cortical map.

The SD is defined by the same formula as the MLD (1), but $d_j$ is now the distance between the *j*-th voxel and the peak of the cortical map, used as a reference point instead of the ECD location. It has been used to quantify the spatial dispersion of each cortical map, independently on whether the latter got close to the corresponding ECD location. As for the MLD, the SD can only be computed for SESAME and wMNE.

For each patient, these three metrics have been applied to cortical maps and ECDs resulting from the analysis of each single epileptic spike, and then averaged across all IEDs.

Finally, the AUC is a global measure of discrepancy between the set of all ECDs and the averaged cortical maps, hence only suited for SESAME and wMNE. It has been computed as follows: first, those voxels in the map whose value is above a given threshold have been defined as “active”, and the remaining ones as “inactive”; we then counted the ECDs located in active voxels as “true positives”, the active voxels in which no ECD has been fit as “false positives”, the inactive voxels in which no ECD has been fit as “true negatives”, the ECDs located in inactive voxels as “false negatives”, and computed the Receiver Operating Characteristic (ROC) curve as the threshold varied. The area under this curve is the AUC, which represents the quality of the classification in active and inactive regions: a value of the AUC close to one indicates very good classification performance, while a value of the AUC close to 0.5 indicates bad classification performance.

The performance metrics were compared by means of the Mann–Whitney U test (Mann and Whitney 1947), while possible correlation between different measures was assessed through the Spearman’s rank correlation coefficient $\rho$ (Zwillinger and Kokoska 1999). The significance threshold was set to .01. For the calculation of the test statistics and of the corresponding *p*-values, as well as for the computation of the ROC curves described above, we made use of the SciPy library (Virtanen et al. 2019).

### Post-surgical Outcome Prediction

In addition to the metrics described above, we also evaluated the post-surgical outcome prediction power of the single methods. To do so, we first assessed to what extent the cerebral lobes indicated by each method as the IZ were concordant to the ones that were included into the surgical plan, considering five regions in each hemisphere: frontal (F, including frontal cortex and anterior cingulate gyrus), temporal (T, including temporal lobe, insula, hippocampus and amygdala), central (C, including precentral and postcentral gyri), parietal (P, including inferior and superior parietal lobules, precuneus, supramarginal, angular gyri and posterior cingulate gyrus) and occipital (O, including lateral occipital cortex, cuneus and lingual gyrus). To each region we associated a percentage in the following way: for ECD and RAP-MUSIC, we evaluated the percentage of dipoles that were estimated in that particular region; for SESAME we computed, from the averaged cortical map, the percentage of posterior probability in that region; for wMNE the percentage of estimated source intensity. Regions whose percentage was not greater than 10% were not considered.

For each patient and each method, the result was defined to be concordant with the surgical plan whenever the corresponding IZ localization with the highest percentage was included within the set of regions that were selected to undergo surgery.

Post-surgical outcomes were divided into two groups: those belonging to Engel’s class I were called good, while those belonging to an Engel’s class from II to IV were called poor.

With these premises, results were classified with respect to concordance and outcome at 1-year after surgical resection or RF-THC as:

- True positive (TP): in case of concordance with surgery and good outcome;
- False positive (FP): in case of concordance with surgery and poor outcome;
- True negative (TN): in case of discordance with surgery and poor outcome;
- False negative (FN): in case of discordance with surgery and good outcome.

We then determined the localization accuracy of each method by means of the following statistical measures (Sammut and Webb 2011): True Positive Rate (TPR, aka sensitivity), True Negative Rate (TNR, aka specificity), Positive Predictive Value (PPV), Negative Predictive Value (NPV) and the $F_1$-score (F1).

In our context, the TPR measures the proportion of good outcomes for which there is concordance with the surgical plan, the TNR measures the proportion of poor outcomes for which there is discordance with the surgical plan, the PPV indicates how often concordance with surgical plan predicts a seizure-free outcome, and eventually the NPV indicates how often discordance indicates a poor outcome.

The $F_1$-score is the harmonic mean of the PPV and the TPR and is a good choice for the imbalanced classes scenario, as it is ours with 13 good outcomes and 7 poor outcomes; it reaches its best value at 1, while 0 means total failure.

There are multiple reasons why the evaluation of the surgical outcome prediction power from the concordance between the localized IZ and the surgical plan should be attempted with due caution and should be given only a relative meaning, comparing the results of the tested methods to those of ECD fitting. First and foremost, the IZ is not the EZ: as explained in Lüders et al. (2006), the former is usually more extensive than the latter and therefore even if a single IED is localized with high accuracy, this may just determine a portion of the IZ which lies outside the EZ. Secondly, there may be IEDs which are generated in small areas of the cortex and that are invisible to scalp recordings. Lastly, post-surgical seizure freedom not only depends on the correct identification of the EZ but also on whether the surgeon does succeed in cutting all of the connections, which may not be possible in certain situations.

## Results

### Performance Evaluation Results

Table 2 summarizes the numerical results of all the metrics for each patient, averaged across IEDs.

**Fig. 2:**
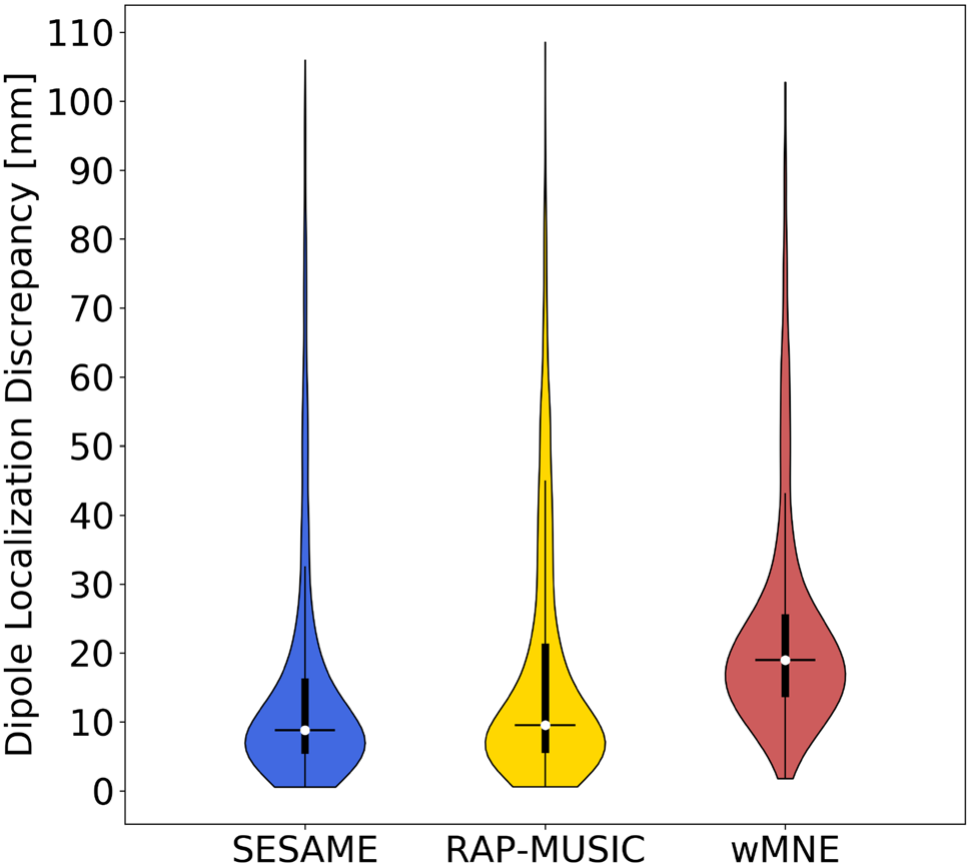
Violin plots of the DLDs across all IEDs and all patients. Despite a seemingly large number of outliers, in 75% of cases the dipole location estimated by SESAME falls within 16.32 mm from the ECD estimated manually; RAP-MUSIC is slightly worse, and wMNE considerably worse

Figures show a certain variability across subjects. For instance, the average DLD—measuring the distance of the point estimate from the corresponding ECD—varies between 7.67 and 33.3 mm in SESAME (mean ± std = 16.33 ± 7.77 mm), between 7.05 and 34.16 mm in RAP-MUSIC (18.03 ± 8.46 mm) and between 14.76 and 38.28 mm in wMNE (23.37 ± 6.77 mm). In Fig. 2 we show the violin plots of the DLD across all IEDs and all subjects (for a total of 1054 IEDs), depicting three distributions with long tail. While the ranges of the three methods are similar, the quartiles indicate that, as expected, the two dipolar methods outperform wMNE, with SESAME providing slightly better results than RAP-MUSIC. In particular, the first three quartiles are: 5.36 mm, 8.78 mm, 16.32 mm for SESAME; 5.51 mm, 9.51 mm, 21.39 mm for RAP-MUSIC; 13.64 mm, 18.96 mm, 25.64 mm for wMNE.

**Table 2:** Performance metrics for each patient, averaged across IEDs

ID	Average DLD (std) [mm]			Average MLD (std) [mm]		Average SD (std) [mm]		AUC	
	SESAME	RAP-MUSIC	wMNE	SESAME	wMNE	SESAME	wMNE	SESAME	wMNE
P1	16.17 (13.56)	18.45 (16.83)	24.24 (7.88)	24.19 (15.58)	52.97 (4.52)	21.73 (14.87)	43.26 (3.77)	0.95	0.82
P2	13.01 (16.27)	18.41 (17.14)	21.02 (11.94)	21.42 (15.21)	52.23 (5.18)	20.72 (12.94)	45.38 (3.87)	0.98	0.89
P3	17.77 (22.44)	20.8 (19.82)	29.37 (15.46)	25.56 (21.3)	64.74 (8.22)	21.61 (19.28)	52.72 (7.22)	0.98	0.88
P4	22.36 (17.59)	27.83 (19.32)	30.71 (23.82)	26.77 (15.98)	60.33 (7.66)	20.83 (13.11)	55.63 (5.29)	0.97	0.97
P5	20.27 (18.83)	25.66 (21.56)	20.63 (11.61)	29.35 (19.97)	57.9 (4.1)	25.1 (13.76)	50.62 (4.88)	0.97	0.93
P6	16.13 (18.58)	20.64 (22.89)	19.18 (7.57)	22.19 (18.75)	55.6 (7.55)	18.18 (13.07)	49.15 (4.02)	0.95	0.96
P7	18.87 (17.12)	20.1 (10.89)	24.83 (12.32)	32.7 (13.33)	56.04 (6.68)	32.65 (11)	53.28 (6.27)	0.81	0.86
P8	32.88 (30.91)	34.16 (27.13)	38.28 (26.75)	41.15 (27.24)	65.7 (5.45)	31.33 (18.46)	54.74 (4.02)	0.78	0.82
P9	8.66 (7.81)	9.87 (8.02)	19.06 (4.74)	12.68 (10.95)	48.17 (5.43)	10.87 (10.36)	39.32 (4.63)	0.98	0.9
P10	7.92 (4.69)	7.05 (4.21)	14.91 (14.9)	11.5 (6.81)	51.99 (6.86)	11.06 (7.97)	49.26 (5.53)	0.96	0.97
P11	21.58 (19.94)	27.46 (21.3)	36.94 (21.96)	27.9 (17.58)	61.78 (8.88)	21.91 (15.21)	53.41 (5.49)	0.86	0.77
P12	9.37 (8.31)	11.77 (10.39)	15.27 (10.67)	12.88 (9.08)	53.11 (4.36)	9.86 (7.6)	50.66 (5.22)	0.98	0.92
P13	15.26 (19.13)	12.83 (12.91)	27.46 (17.25)	17.33 (17.76)	56.59 (5.29)	12.25 (11.97)	53.45 (5.14)	0.95	0.94
P14	31.5 (28.4)	31.42 (28.12)	30.25 (22.75)	36.46 (27.99)	55.18 (7.69)	14.55 (14.75)	47.41 (5.78)	0.9	0.96
P15	11.01 (14.99)	10.35 (14.67)	15.43 (9.98)	12.17 (14.42)	50.11 (5.85)	7.49 (4.27)	44.46 (4.3)	0.99	0.97
P16	13.78 (17.77)	14.54 (18.13)	17.65 (12.12)	15.02 (15.01)	54.57 (7.43)	9.71 (8.53)	48.01 (6.05)	0.99	0.97
P17	33.33 (22.14)	33.45 (21.54)	29.82 (20.42)	34.08 (20.7)	58.98 (8.9)	14.6 (9.51)	53.59 (6.83)	0.9	0.92
P18	12.15 (12.9)	7.42 (5.11)	14.76 (6.22)	13.04 (11.41)	44.27 (5.05)	11.41 (11.65)	41.97 (7.42)	0.98	0.95
P19	9.56 (6.28)	11.69 (8.32)	18.96 (11.47)	12.9 (7.02)	52.49 (5.18)	10.66 (7.54)	48.43 (5.71)	0.96	0.92
P20	11.6 (10.86)	12.52 (11.23)	25.14 (15.71)	14.02 (10.36)	53.32 (6.07)	11.2 (8.3)	49.11 (8.28)	0.91	0.87
P21	7.67 (6.37)	9.22 (8.65)	18.75 (8.33)	10.04 (5.76)	55.59 (6.33)	8.11 (5.14)	49.28 (5.44)	0.99	0.97
P22	8.45 (5.62)	11.08 (9.43)	21.6 (17.19)	12.07 (6.74)	57.75 (6.06)	10.77 (7.7)	52.65 (6.28)	0.96	0.93

The average MLD shows that the probability map of SESAME is much closer to the ECD locations than the intensity map of wMNE (21.15 ± 9.21 mm vs 55.43 ± 4.93 mm; U = 0, *p*<.01).

As expected, the average SD of SESAME is significantly lower than the one of wMNE (16.21 ± 7.15 mm vs 49.35 ± 4.26 mm; U = 0, *p*<.01); correlation between the average SD and the average DLD holds both for SESAME ($\rho =$0.77, *p*<.01) and for wMNE ($\rho =$0.61, *p*<.01): this indicates that, when the uncertainty is small, the results also tend to agree more with those of dipole fitting.

The average SD is significantly similar to the average MLD for SESAME (16.21 ± 7.15 mm vs 21.15 ± 9.21 mm; U = 147, *p* = .026), not for wMNE (49.35 ± 4.46 mm vs 55.43 ± 4.93 mm; U = 90, *p*<.01); this confirms that wMNE maps are centered in locations that are further from those of ECD wrt SESAME maps.

In comparison with wMNE, SESAME provides a greater or equal value of the AUC in sixteen subjects out of twenty-two (U = 335, *p* = .015); in addition, the five largest differences in absolute value are all in favour of SESAME. Eventually, the average AUC of SESAME is 0.94, while the average AUC for wMNE is 0.91. These results indicate that not only the dipole locations and the cortical maps computed by SESAME are closer to the ECD locations (as shown by the discrepancy measures above), but also that the high-probability regions of SESAME actually *hit* the ECD locations more often than the high-intensity regions of wMNE do. We also notice that the AUC of SESAME is either very high or, in few cases, relatively low, because of the focal nature of the probability maps estimated by the method; on the other hand, the AUC of wMNE features more uniformly distributed values, as a consequence of the smoothness of the estimated cortical maps.

**Fig. 3:**
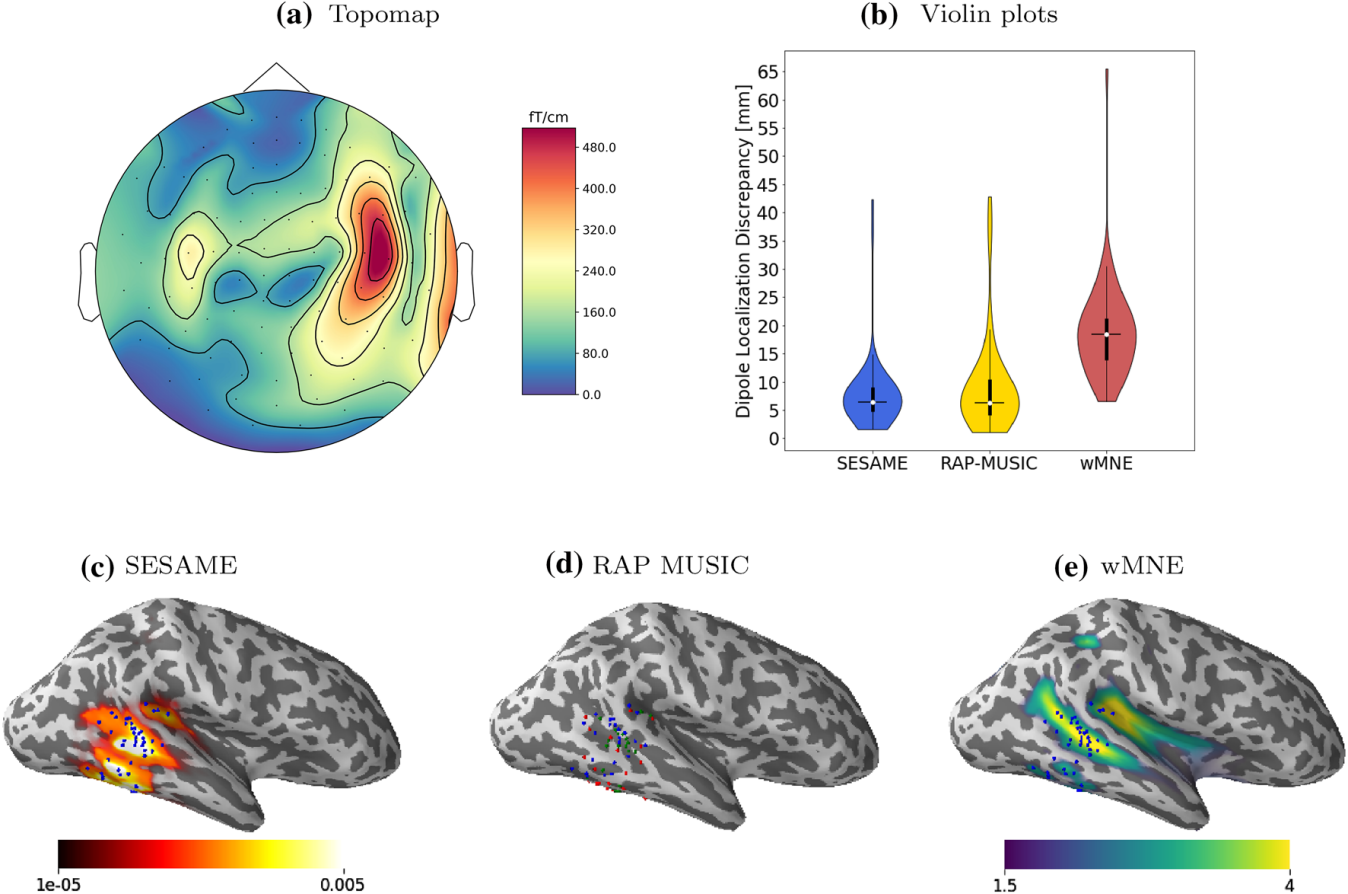
Analysis of patient P21. The figure shows: the spatial topography corresponding to the peak of one of the selected IEDs (**a**); the violin plots of the DLDs (**b**); the color-coded cortical maps of SESAME (**c**) and wMNE (**e**), averaged across all spikes, with the ECD locations (blue dots) superimposed; the dipole locations estimated by RAP-MUSIC (**d**, red dots), also with ECD locations (blue dots) superimposed; green dots indicate coincidences

**Fig. 4:**
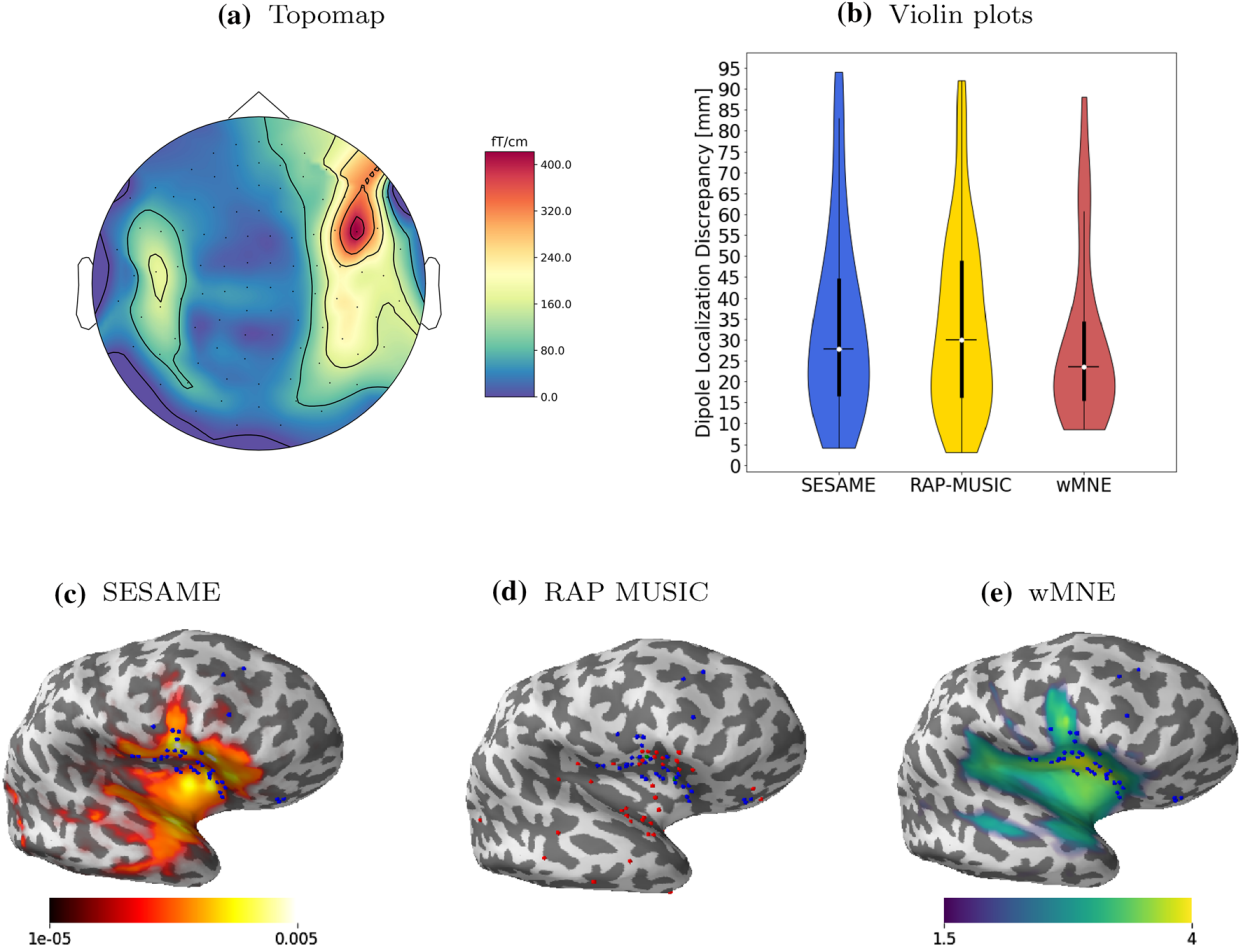
Analysis of patient P17. The figure shows: the spatial topography corresponding to the peak of one of the selected IEDs (**a**); the violin plots of the DLDs (**b**); the color–coded cortical maps of SESAME (**c**) and wMNE (**e**), averaged across all spikes, with the ECD locations (blue dots) superimposed; the dipole locations estimated by RAP-MUSIC (**d**, red dots), also with ECD locations (blue dots) superimposed; green dots indicate coincidences

In Figs. 3 and 4 we provide a visual representation of the global assessment of the irritative zone as provided by ECD fitting analysis and by the three automatic methods, in two selected patients. Specifically, we chose P21 and P17 as representative of the best and of the worst case, respectively, as measured by the DLD of SESAME. In P21 we observe that SESAME appears to cover the areas corresponding to ECD locations more uniformly than wMNE does, and similarly to RAP-MUSIC; this is confirmed by the violin plots. On the other hand, in P17 all three automatic methods localize the majority of inter-ictal epileptic activity in the temporal lobe, while most of the ECD locations belong to the frontal lobe.

### Post-surgical Outcome Prediction Results

In Table 3 we report the clinical indication provided by all four methods in terms of cerebral lobes. SESAME localization of the IZ at a lobar level turned out to be extremely similar to that of ECD fitting; the mode of the distribution was equal in all subjects but two (P2 and P17), for whom in fact the lobe indicated by SESAME was the same where patients underwent surgery, and with good outcome.

**Table 3:** Localization of the IZ provided by all four methods in terms of cerebral lobes

ID	ROI Lobar (>10%)			
	ECD	SESAME	RAP-MUSIC	wMNE
P1	R F (39%), R C (31%), R P (25%)	R F (36%), R C (23%), R P (21%), R T (11%)	R F (53%), R C (19%), R P (14%)	R F (18%), R T (18%), R P (14%), L T (12%)
P2	L C (53%), L P (43%)	L P (41%), L C (29%), L F (13%)	L P (47%), L C (30%)	L P (17%), L T (14%), R P (13%), L F (11%)
P3	L T (72%), L C (15%), L P (11%)	L T (56%), L C (19%), L P (13%)	L T (61%), L C (20%)	L T (20%), L P (12%), L F (12%), R T (11%)
P4	R T (75%)	R T (61%), R F (23%)	R T (49%), R F (18%), R O (12%)	R T (19%), R F (16%), L T (14%), R P (11%)
P5	R P (50%), R T (33%), R C (17%)	R P (34%), R T (34%)	R P (39%), R T (39%), R O (11%)	R T (21%), R P (16%), L T (12%), R F (12%)
P6	R C (56%), R P (20%), R F (15%)	R C (45%), R F (16%), R P (15%)	R C (46%), R F (17%), R P (15%)	R F (14%), R P (13 %), R T (13%), L T (13%), L F (13%), L P (11%)
P7	L T (50%), L P (50%)	L P (38%), L T (29%), L F (15%)	L P (50%), L T (38%), R P (12%)	L T (19%), L P (14%), L F (13%), R T (11%), R F (11%)
P8	L T (57%), L P (21%)	L T (26%), L P (16%), L C (14%), R P (14%), R C (11%)	L P (29%), R P (21%), L T (14%), L F (14%)	L T (16%), R T (15%), L F (13%), R F (12%)
P9	R P (75%), R C (23%)	R P (71%), R C (21%)	R P (71%), R C (23%)	R P (22%), R T (14%), R F (11%)
P10	R C (47%), R P (44%)	R C (49%), R P (41%)	R C (49%), R P (47%)	R P (18%), R T (15%), L T (12%), R F (11%)
P11	L F (35%), R F (16%), L T (13%), L P (12%)	L F (29%), L T (15%), R F (14%), L P (12%)	L T (25%), R F (23%), L F (17%)	L P (14%), L F (13%), R P (13%), L T (13%), R T (12%), R F (12%)
P12	R T (60%), R F (17%), R C (14%)	R T (59%), R F (18%), R C (11%)	R T (60%), R F (17%), R P (11%)	R T (19%), R P (14%), R F (14%), L T (11%)
P13	L T (85%), L O (11%)	L T (75%), L P (11%)	L T (79%), L O (11%)	L T (20%), L P (16%), R T (12%)
P14	L T (88%), L P (12%)	L T (47%), L P (24%), R P (15%)	L T (47%), L P (29%), R P (18%)	L T (19%), L P (16%), R P (12%), R T (11%)
P15	R P (72%), R C (21%)	R P (72%), R C (19%)	R P (82%), R C (13%)	R P (22%), R T (14%), L P (11%), R C (11%), R F (11%)
P16	R T (98%)	R T (84%)	R T (89%)	R T (24%), R P (15%), R F (12%)
P17	R F (54%), R T (27%), R C (17%)	R T (47%), R F (37%)	R T (50%), R F (35%)	R T (20%), R P (15%), R F (14%)
P18	R P (67%), R T (25%)	R P (64%), R C (16%), R T (16%)	R P (75%), R T (17%)	R P (25%), R T (15%)
P19	R T (65%), R P (25%)	R T (61%), R P (25%)	R T (60%), R P (25%), R O (12%)	R T (19%), R P (18%), L P (12%)
P20	L T (42%), L P (25%), R P (12%)	L T (40%), L P (20%), R P (13%), L C (12%)	L T (40%), L P (19%), R P (17%), L C (12%)	L T (17%), L P (17%), L F (12%)
P21	R T (97%)	R T (92%)	R T (95%)	R T (22%), R P (18%), R F (11%)
P22	L T (83%)	L T (77%), L P (11%)	L T (78%)	L T (22%), L P (13%)

*L* left, *R* right, *F* frontal, *C* central, *P* parietal, *T* temporal, *O* occipital

Concomitantly, the mode of the cerebral lobe distribution provided by RAP-MUSIC differed from that of ECD fitting four times (P2, P8, P11 and P17), of which P2 and P17 are concordant with SESAME, while P8 and P11 provide indications in disagreement with both SESAME and surgery. As for wMNE, there are six subjects in which the mode of the distribution is different from ECD fitting (P2, P5, P6, P10, P11 and P17). Again, only P2 and P17 are in accordance with SESAME while, among the other cases, only P6 agrees with surgery.

Some cases deserve to be analyzed individually, namely: P4 and P5 for the localization of the IZ contralateral to MRI, P7 for its high SD, P8, P14 and P17 for their high DLD.

P4 underwent surgery which led to Engel class 4. We can hypothesize that this patient had a wide epileptogenic network which was underestimated by the routine diagnostic work-up. All of the methods localized the IZ contralaterally with respect to the area indicated by MRI, being wMNE the only one which included the left hemisphere in its highly dispersed solution. The discordance between MRI and MSI could have suggested a more thorough evaluation before surgery.

The results of P5 are very similar to those of P4, with the only crucial difference that, in this case, the post-surgical outcome was good. We can speculate on the number of IEDs selected by the neurophysiologist which was small because of the presence of confounding artifacts. Anyway, even though for the purpose of this work we can observe that the solutions proposed by the automatic methods showed to be comparable with the one obtained by ECD fitting, this patient represented a failure for MSI as a whole.

In P7 SESAME yields the highest SD, which indicates that localization of individual IEDs is highly uncertain. This may be due either to lower SNR of the data, compared to other patients, or to a less focal structure of the activation. We also notice that, unfortunately, the outcome of surgery was not satisfactory in this case.

In P8 SESAME has the second highest DLD and also the second highest SD; for this subject, who was diagnosed with a bilateral ulegyria, all ECDs are fit in the left hemisphere, while all automatic methods present a more complex and uncertain solution in which brain activity is also detected in the right hemisphere (where surgery was actually performed, with good outcome), thus adding to the hypothesis of a strong bias introduced by channel selection in the ECD fitting analysis.

In P14 SESAME shows the third highest DLD. As for P8, this is likely due to the fact that all ECDs belong to the left hemisphere, while all automatic methods localize some of the IED generators in the right hemisphere. The source dispersion, however, is here considerably smaller, indicating good confidence in the localization in both hemispheres.

Finally, in P17, SESAME presents the highest DLD and—as in P14—a not particularly high SD, indicating again good confidence in the results. As discussed above, in this case all automatic methods agree in pointing out the temporal lobe as the most probable IZ, in disagreement with ECD fitting but in concordance with the surgery plan which led to seizure freedom.

**Fig. 5:**
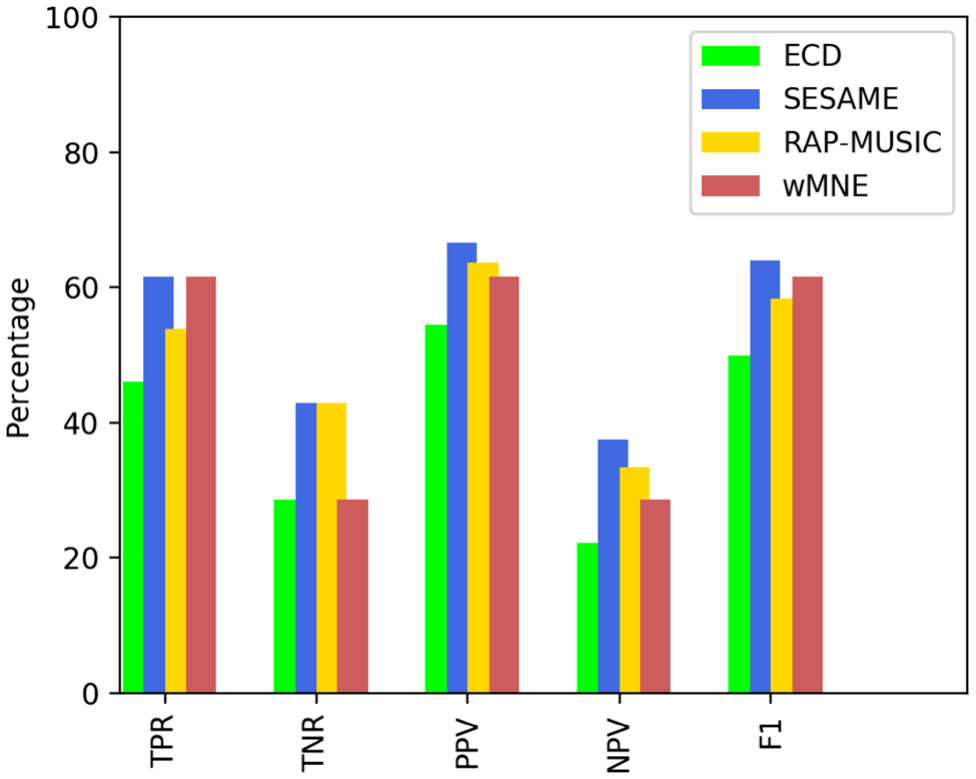
Statistical measures for the classification problem set up in Sect. 2.5

To conclude the Section, in Table 4 and in Fig. 5 we provide the confusion matrices and the statistical measures respectively that describe the performance of the four algorithms in the binary classification problem set up in Sect. 2.5.

**Table 4:** Confusion matrices for the classification problem set up in Sect. 2.5

Outcome	Concordance	
	Yes	No
*ECD fitting*		
Good	6	7
Poor	5	2
*SESAME*		
Good	8	5
Poor	4	3
*RAP-MUSIC*		
Good	7	6
Poor	4	3
*wMNE*		
Good	8	5
Poor	5	2

We observe that all the automatic methods perform better than ECD fitting and that SESAME is the one that features the best performance in all the measures.

## Discussion

The correct localization of the epileptogenic zone represents the best prognostic factor in the pre-surgical evaluation of patients with drug-resistant focal epilepsy. Although invasive SEEG recordings are still mandatory in cases in which routine electro-clinical investigations present discrepancies and/or structural MRI is negative, the use of non-invasive functional neuroimaging techniques is expected to be useful to prevent unnecessary surgery and/or to guide invasive recordings (Baroumand et al. 2018). In this context, MEG seems promising since it enables the analysis of the whole brain electromagnetic activity with an excellent temporal resolution combined with a good spatial resolution. However, common usage of MEG data for the identification of the epileptogenic zone has often the major drawback of involving subjective choices. For example, to increase SNR, the source modeling is most widely performed by fitting ECDs from a subset of sensors whose selection is made at the examiner’s discretion.

In virtue of its clinical added value (De Tiège et al. 2012; Duez et al. 2019), magnetic source imaging is part of the pre-surgical evaluation in an increasing, albeit still limited, number of epilepsy centers worldwide (Mouthaan et al. 2016). However, no standardized approach in the localization of the irritative zone exists: each center takes its own choice on using a head model based on a template MRI or on the patient’s specific MRI and there is not a standard way to perform source modeling. In this connection, exploiting an automated localization method in the analysis pipeline could, on the one hand, widen the use of magnetic source imaging as it would not be necessary to acquire specific and complex skills, and, on the other hand, ensure the reproducibility and comparability of the results.

The primary aim of this retrospective study was to investigate whether, and to what extent, traditional ECD fitting can be replaced by an automatic and objective procedure; in particular, we were interested in validating a recently proposed Bayesian dipole modeling algorithm—called SESAME (Sorrentino et al. 2014; Sommariva and Sorrentino 2014)—in the task of localizing the irritative zone. To this aim we performed source modeling on single interictal epileptiform discharges from twenty-two patients, analyzing over a thousand topographies; we used the results of an ECD fitting analysis carried out by an expert user as a benchmark. In addition and for comparison, we also performed source modeling with two widely used algorithms, RAP-MUSIC and wMNE. The validation involved both patients whose MRI revealed the presence of a cortical lesion and patients having a negative MRI.

The results are encouraging, although they must be confirmed by further prospective studies on larger cohort of epileptic patients. Even in the localization of single IEDs, where SNR is typically rather low, the dipole localization discrepancy from the ECD fitting solution was below 1.63 cm in 75% of cases with SESAME, below 2.14 cm for 75% of reconstructions with RAP-MUSIC and lower than 1.36 cm in only 25% of the results with wMNE. Nonetheless, drawing conclusions from the analysis of a single IED would be a risky affair due to the presence of some highly discrepant elements, appearing as outliers and going up to 10 cm of distance. This fact is quite natural, considering that ECD locations are obtained by an experienced user with channel selection, while the automatic methods were applied here to the whole topographies: in the case of complex activation patterns, this can make a huge difference. However, when looking at the big picture in which a relatively large number of IEDs has been taken into account, the impact of these outliers was reduced to an almost negligible effect.

In the majority of cases, the three tested methods showed good agreement both with ECD fitting analysis and among themselves. In particular, wMNE yielded—as expected—the most discrepant results with respect to dipole fitting, while SESAME is the one that got closer and, to some extent, also provided an indication of the reliability of the solution itself. In a very small number of cases discrepancy was high; in those cases, however, we presented elements not to fully trust the ECD fitting localization.

The irritative zone, as identified by ECD, was often less extended than those determined by the other methods; this is reasonable, in the light of the fact that the epileptologist is likely to use some form of prior information in his/her analysis, particularly in the channel selection step. On the other hand, SESAME and RAP-MUSIC results were consistently very close to those of ECD in terms of lobar percentages, while wMNE provided considerably more widespread solutions and therefore a more vast irritative zone.

In the binary classification problem, based on the concordance between the localization of the irritative zone and the surgical plan and on the post-surgical outcome at least one year after surgery, the four methods performed similarly, with SESAME leading the group. In particular, concordance between SESAME localization and the surgical plan showed to be a good predictor of seizure freedom, even if, at the current stage, results must be taken with due caution. On this account, a more definitive assessment is being considered as a future work, involving a larger cohort of subjects and possibly evaluating the concordance with the surgical plan at a sublobar level.

## Conclusion

Pre-surgical localization of the epileptogenic zone from MEG data is largely accomplished using Equivalent Current Dipole fitting analysis, a procedure that involves subjective choices and requires expertise. In this study we applied automated source localization algorithms to MEG data from twenty-two epileptic patients, with the aim of making the source localization process more objective. We compared three publicly available methods (SESAME, RAP-MUSIC and wMNE) in the task of localizing the generators of single interictal epileptiform discharges. We compared their results with those obtained by ECD fitting analysis made by an expert epileptologist. The three methods provided fairly good results, with some marked differences among them. The results of SESAME were most similar to those of the ECD fitting analysis, with a median distance of 9 mm (RAP-MUSIC: 11 mm; wMNE: 16 mm), and with 75% of the reconstructions falling within 1.6 cm (RAP-MUSIC: 21 mm; wMNE: 26 mm) from the corresponding ECD. All methods presented a relatively large number of outliers; however, the overall assessment of the irritative zone, computed through averaging across localization maps of multiple interictal epileptiform discharges, was often similar to that provided by ECD fitting analysis. Using the lobar-level information from the surgery plan and that from the 1-year outcome of the surgery, we performed an analysis of the predictive power of the methods, where SESAME obtained the highest score, and ECD the lowest.

In conclusion, our results seem to indicate the feasibility of replacing manual dipole fitting with automated methods in the source modeling step of the pre-surgical localization of the irritative zone, thus making the entire process more objective.
